# The Asian Correction Can Be Quantitatively Forecasted Using a Statistical Model of Fusion-Fission Processes

**DOI:** 10.1371/journal.pone.0163842

**Published:** 2016-10-05

**Authors:** Boon Kin Teh, Siew Ann Cheong

**Affiliations:** 1 Division of Physics and Applied Physics, School of Physical and Mathematical Sciences, Nanyang Technological University, 21 Nanyang Link, Singapore 637371, Republic of Singapore; 2 Complexity Institute, Nanyang Technological University, Block 2 Innovation Centre, Level 2 Unit 245, 18 Nanyang Drive, Singapore 637723, Republic of Singapore; University of Rijeka, CROATIA

## Abstract

The Global Financial Crisis of 2007-2008 wiped out US$37 trillions across global financial markets, this value is equivalent to the combined GDPs of the United States and the European Union in 2014. The defining moment of this crisis was the failure of Lehman Brothers, which precipitated the October 2008 crash and the Asian Correction (March 2009). Had the Federal Reserve seen these crashes coming, they might have bailed out Lehman Brothers, and prevented the crashes altogether. In this paper, we show that some of these market crashes (like the Asian Correction) can be predicted, if we assume that a large number of adaptive traders employing competing trading strategies. As the number of adherents for some strategies grow, others decline in the constantly changing strategy space. When a strategy group grows into a giant component, trader actions become increasingly correlated and this is reflected in the stock price. The fragmentation of this giant component will leads to a market crash. In this paper, we also derived the *mean-field market crash forecast equation* based on a model of fusions and fissions in the trading strategy space. By fitting the continuous returns of 20 stocks traded in Singapore Exchange to the market crash forecast equation, we obtain crash predictions ranging from end October 2008 to mid-February 2009, with early warning four to six months prior to the crashes.

## Introduction

Today, the financial markets have become globally interconnected. Market players frequently maintain portfolios over many asset classes as an insurance against idiosyncratic risks. This raises concerns about susceptibility to systemic risks, and consequently the sustainability of the financial systems. This is worrisome, particularly for the central banks, regulators, and financial institutions. The Global Financial Crisis of 2007-2008 wiped out 34 trillion US dollars of value across financial markets around the world [1]. This is equivalent to the combined GDPs of the United States (US$17.42 trillions [2]) and the European Union (US$18.45 trillions [3]) in 2014. The defining moment of this crisis was the failure of Lehman Brothers in September 2008, which sent shock waves through financial markets around the world in the form of global market crashes namely the October 2008 crash and the Asian Correction (March 2009).

Had the Federal Reserve seen these crashes coming, they might have worked harder to bail out Lehman Brothers, and prevented the crashes altogether. Financial economists largely believe that financial crises are due to exogenous factors, and are hence not predictable. We feel that this opinion reflects more on the limitations of the models chosen by the economic community, rather than the task being fundamentally impossible. For instance, statistically stationary model [4–6] cannot produce booms and busts, whereas time series model with stochastic jumps [7–9] produces unpredictable booms and busts. Beyond statistical modeling, Palmer et al. introduced in 1994 the Santa Fe Stock Market Model [10], and showed that the artificial market can crash in the absence of exogenous shocks. We expect precursory signatures to exist for the endogenous booms and busts in the Santa Fe Stock Market Model, but no one has looked into this systematically. Alternatively, we can also try model-free forecasting of extreme market events using only empirical data [11]. The most successful of these is the *Log Periodic Power Law* (LPPL) method [12, 13], which was originally developed by Didier Sornette in 1996 [14] to forecast earthquakes. As many have liken financial crashes to earthquakes [15–17], Johansen and Sornette fitted the LPPL to stock market indices, and found that the real market crashes times are very close to the finite-time singularities of the fitted LPPL. More importantly, they concluded that 25 out of the 49 crashes identified exhibit LPPL signatures and thus be classified as endogenous crashes [18].

Inspired by a Soup-of-Groups (SOG) model description of statistical fusion-fission processes in fault planes, Cheong et al. were able to forecast the timings, magnitudes, and locations of large Taiwanese earthquakes [19]. Furthermore, in a recent paper [20, 21], we tracked robust clusters of stocks in the Singapore Exchange over 2008 and 2009, and found that these routinely merged to form larger clusters, and also disintegrated into smaller clusters. In particular, one of the clusters grew steadily into a giant cluster as we approached the October 2008 crash. As stock price movement is governed by market participants, this trading strategy clusters picture is similar to the “opinion convergence and divergence” proposed by Lin et al. [22]. Market participants read and react to market signals, during normal period they have diverse opinions, but they behave coherently when there are large price swings. These findings demonstrates that a financial market naturally self-organizes into clusters of strongly-correlated stocks (and trading strategy with similar opinions) that undergo fusions and fissions. Due to the coexistence of fusion-fission processes in both fault planes and trading strategies space, we wanted to duplicate our success with forecasting earthquakes to forecast financial crashes using the SOG model.

## The Soup-of-Groups Model

The SOG model was first introduced by Neil Johnson et al. to study the emergent behavior in a vastly different complex systems. It was first applied to model the underlying dynamics of human insurgency [23], where terrorists are assumed to form groups that merge with each other in preparation for attacks, and disintegrate after a successful attack or to avoid detection by security forces. By comparing the model against a database of past terrorist attacks, Johnson et al. found that the terrorist groups are equally effective, and the number of casualties depends only on the group size, which is distributed as a power law with exponent *α* = 2.5. In the follow up paper [24] they suggested that the escalation rate and timing of the fatal attacks from terrorists follow naturally from SOG dynamics. In addition, Johnson et al. also tried to explain contagion dynamics on social networks in terms of the SOG model, particularly in the intermediate regime that relates individual behaviors to social group structures [25].

**Fig 1:**
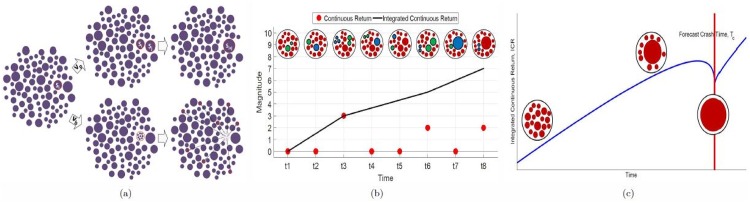
(a) Illustration of Soup-of-Groups (SOG) model, as a system of clusters with various sizes where clusters can undergo fusion at a rate of *ν*_*p*_ or fission at a rate of *ν*_*b*_. The upper half represents the fusion process as two clusters with size *s*_*i*_ and *s*_*j*_ are selected and fused into a larger cluster with size *s*_*i*_ + *s*_*j*_. In contrast, during the fission process (lower half) the selected cluster with size *s*_*i*_ will experience complete fragmentation into *s*_*i*_ size−1 clusters. (b) A schematic showing how SOG fusion and fission processes gives rise to Continuous Return (*CR*) events. Here, the selected cluster is colored green and the resultant clusters after fusion or fission process are colored blue. Each *CR* is due to a cluster fragmentation with a magnitude proportional to the cluster size. In contrast, a fusion process generates no *CR* but makes the cluster bigger, and possibly into a giant cluster. In (c) we show the curve generated by Eq (2) that illustrates how the Integrated Continuous Return (*ICR*) change when a giant cluster is present and growing. Initially, in the absent of giant cluster, the *ICR* grows linearly due to fragmentation of clusters with an equilibrium distribution of sizes. However, when a giant cluster forms at the expense of other clusters, we start to get only fragmentations of smaller clusters and hence a slow down in *ICR* growth. Finally at *t*_*c*_ where the giant cluster has exhausted all the resources, and reached the maximum allowed size, it must fragment completely in the next time step.

The SOG model, as the name suggests, consists of a “soup” (system) of “groups” (clusters) with various sizes. The essence of SOG dynamics is the fusion-fission processes among clusters. For instance, a cluster of size *s*_*i*_ can merge with a cluster of size *s*_*j*_ to give a cluster with size *s*_*i*_ + *s*_*j*_ at a rate of *ν*_*p*_, or a cluster of size *s*_*k*_ can fragment completely into *s*_*k*_ clusters with size 1 at a rate of *ν*_*b*_, see Fig 1(a) for illustration and refer to the S2 File for more details. In an infinite element equilibrium system, Neil Johnson et. al. discovered that the equilibrium distribution for the SOG model is an *Exponential Truncated Power Law* (ETPL) distribution [26] as

$$\begin{array}{c} N_{s}=f\left(S\right)∼s^{-\frac{5}{2}}\exp\left(-S_{o}s\right);\;\;S_{o}=-\ln(\frac{4\left(\nu_{p}+\nu_{b}\right)\nu_{p}}{{\left(2\nu_{p}+\nu_{b}\right)}^{2}}). \end{array}$$

## SOG Forecasting

In finance, one of the most widely used definition for quantifying financial returns is the simple return (and log return). However, the distribution of financial returns have been found to depend on the choice of time horizon, making such returns ambiguous [27]. To get around this ambiguity, we introduce *continuous returns* defined as the continuous price movement in the same direction (refer to the subsection Continuous returns in the Data and Methods section). This definition of an event agrees with that used by da Cruz and Lind [28]. In financial markets, market participants are heterogeneous, so even though they read the same market signals they may arrive at different trading strategies. However, if the market signal is strong, these traders may arrive at the same trading strategies, thereby reinforcing the market signals, leading to the formation of strategies blocks. When a strategy block is executed, market participants within the strategy block will react in the same way, hence generating a continuous return with a magnitude that depends on the size of the strategy block.

In this study, we focus on data from the Singapore Exchange (SGX) because we are familiar with stocks listed in the SGX. The SGX is also strongly coupled to financial markets around the world, and readily reflects the global market movement. We therefore test the SOG forecasting method for 20 component stocks of the Straits Times Index (STI) over the period January 2006 to December 2011, which is approximately three years before the October 2008 crash and three years after the October 2008 crash (read subsection Data in section Data and Methods). Since the SOG dynamics is universal, we expect it to be able to do forecasting with the model even when the stock price dynamics is only approximately SOG. To demonstrate this approximate SOG behavior we check the distribution of the continuous returns against the ETPL distribution. For comparison, we also fit the empirical data to the power law (PL) and asymptotic exponential (EXP) distribution using the method developed by Clauset et al. to estimate these parameters (${\hat{x}}_{min}$, $\hat{\alpha }$, and $\hat{\beta }$) [29]. A detail description of the fitting procedure can be found in the S4 File. The fitting results from section *Distribution of Continuous Returns* in S3 File indicate that the continuous returns for 20 stocks are best fitted to the ETPL, as the PL tends to decay slower than the data, while EXP tends to decay faster than the data. Overall, the average ${\hat{\alpha }}_{ETPL}$ and ${\hat{\beta }}_{ETPL}$ across all stocks is 1.82 ± 0.23 and 0.37 ± 0.08 respectively. The reported ${\hat{\alpha }}_{ETPL}$ is lower when compared with the SOG exponent of *α* = 2.5. Even though we can assume that the continuous return is directly proportional to the size of the cluster, yet the result shows this relationship does not hold. But, if we assume that a cluster of size *s* produces a continuous return of *s*^*ζ*^, where *ζ* = 2.5/*α*, then the SGX will indeed be a SOG system. We will use this modified scaling to formulate the forecasting equation (Eq (2)).

In the SOG model each continuous return is caused by a fragmentation of the strategy block, therefore a market crash is associated with the fragmentation of a giant cluster. Fig 1(b) illustrates the relation between the continuous return and the SOG fusion and fission process. During the fusion process two clusters merge and form a larger cluster, but generate no continuous return. On the other hand, each fission process will generate a continuous return. In these descriptions, a market crash is due to the fragmentation of a giant cluster (giant strategy block) that rapidly drives the price downwards. Thus if we can track the size and growth of the giant cluster we should be able to deduce the time when the market will crash. This can be done by determining the time when the giant cluster will reaches its maximum size. This time acts as the upper bound time for fragmentation of the giant cluster, thus the forecasted crash time. Unfortunately, we cannot directly observe the giant cluster size in the financial market, since we cannot simultaneously track the opinions and strategies of all market participants. Instead, we infer the giant cluster size based on the continuous returns, which becomes smaller as the giant cluster grows bigger and exhausts the larger clusters around it. This manifests itself as a slowing down in the growth of the integrated continuous returns.

In order to build the forecasting model, the inputs are: the size of the giant cluster *s*_*G*_(*t*), the size distribution of the rest of clusters *f*(*s*), as well as the relations between the magnitude of the continuous return and the cluster size *CR*_*t*_ = *CR*_*t*_(*s*_*t*_). We make several assumptions regarding these inputs. First, we assume that the giant cluster grows linearly with time as *s*_*G*_(*t*) = *γt*. Second, instead of using the ETPL distribution, which is difficult to work with, we use the simplified PL distribution *f*(*s*) = *As*^−2.5^. Lastly, we assume that the continuous return is proportional to the cluster size, given as $CR_{t}\propto s_{t}^{\zeta }$ in order for the dynamics of continuous return to be approximately SOG. Based on these assumptions, we can write down the expected integrated continuous return $\hat{ICR}\left(0,t\right)$ from *time* = 0 to *time* = *t* as

$$\begin{array}{ccc} \hat{ICR}\left(0,t\right) & = & \int_{0}^{t}dt\int_{s_{o}}^{s_{max}-s_{G}\left(t\right)}dsAs^{\zeta }f\left(s\right)=\int_{0}^{t}dt\int_{s_{o}}^{s_{max}-s_{G}\left(t\right)}dsAs^{\zeta -2.5}\\ & = & \frac{A}{\zeta -1.5}\{\frac{1}{\gamma (0.5-\zeta )}[{\left(s_{max}-\gamma t\right)}^{\zeta -0.5}-s_{max}^{\zeta -0.5}]-s_{o}^{\zeta -1.5}t\}. \end{array}$$ (1)

Assuming the giant cluster started growing at *t*_*o*_, Eq (1) becomes

$$\begin{array}{ccc} & & \hat{ICR}\left(t_{o},t\right)=\\ & & \;\frac{A}{\zeta -1.5}\{\frac{1}{\gamma (\zeta -0.5)}[{\left(S_{max}-\gamma \left\{t-t_{o}\right\}\right)}^{\zeta -0.5}-S_{max}^{\zeta -0.5}]-S_{o}^{\zeta -1.5}\left\{t-t_{o}\right\}\}+ICR_{o}. \end{array}$$ (2)

Eq (2) is a mean-field forecasting equation that we show in Fig 1(c). From Fig 1(c), we observe that before the giant cluster started growing, $\hat{ICR}\left(t\right)$ grows linearly with time. As the giant cluster grows, the growth rate of $\hat{ICR}\left(t\right)$ decreases. More importantly, a singularity occurs when the giant cluster reaches the maximum size of *S*_*G*_(*t*_*c*_) = *S*_*max*_ = *γ*(*t*_*c*_ − *t*_*o*_). We call this time the *forecasted crash time* (*t*_*c*_) which is

$$\begin{array}{c} t_{c}=\frac{S_{max}}{\gamma }+t_{o}. \end{array}$$ (3)

From the data, we define the empirical *integrated continuous returns* from *t*_*Start*_ to *t*_*End*_ as

$$\begin{array}{c} ICR\left(t_{Start},t_{End}\right)=\sum_{k=i;\;t\left(i\right)\geq t_{Start}}^{N;\;t\left(N\right)\leq t_{End}}CR_{t(k)}, \end{array}$$ (4)

which is the cumulative sum of the continuous returns. We fit the empirical integrated continuous returns *ICR*(*t*_*Start*_, *t*_*End*_) to our forecasting model $\hat{ICR}\left(t_{Start},t_{End}\right)$ (Eq (2)) to obtain the forecasted crash time *t*_*c*_. For the fitting, we use the non-linear least square method in MATLAB to estimate the parameter set that minimizes the overall residuals. In practice, we must only use data available up to the present moment. Therefore to mimic a real-time forecasting using historical data, we work with dynamic fitting windows starting at *t*_*Start*_ and ending at *t*_*End*_, where *t*_*End*_ must be before the market crash we want to ‘forecast’. To do this, we fix a *t*_*Start*_ then we start with *t*_*End*_ one month forward and increase *t*_*End*_ 2 weeks at a time. We then obtain *t*_*c*_ as a function of *t*_*End*_ for a given *t*_*Start*_, *t*_*c*_ = *t*_*c*_(*t*_*End*_|*t*_*Start*_).

## Results

Our objective is to use the SOG model to detect early warnings of market crashes by forecasting the crash times of the STI component stocks. This is a different approach compared to traditional finance and econometric models, wherein sudden changes are assumed to be brought about by exogenous shocks. In the SOG model, a market crash is endogenous, driven by the giant cluster formation and its fragmentation. Within the period that we study, from Jan 2006 to Dec 2011, two global financial crashes left their marks on the SGX. As shown in S Fig 1 in S1 File, the October 2008 crash and the Asian Correction that caused the STI to tumble to its lowest at 1600.29 (27 Oct 2008, October 2008 crash), with a slight recovery after that and further sliding to 1456.29 (9 Mar 2009, Asian Correction). The first crash was larger, thus we select 27 Oct 2008 as our reference date for the market crash.

**Fig 2:**
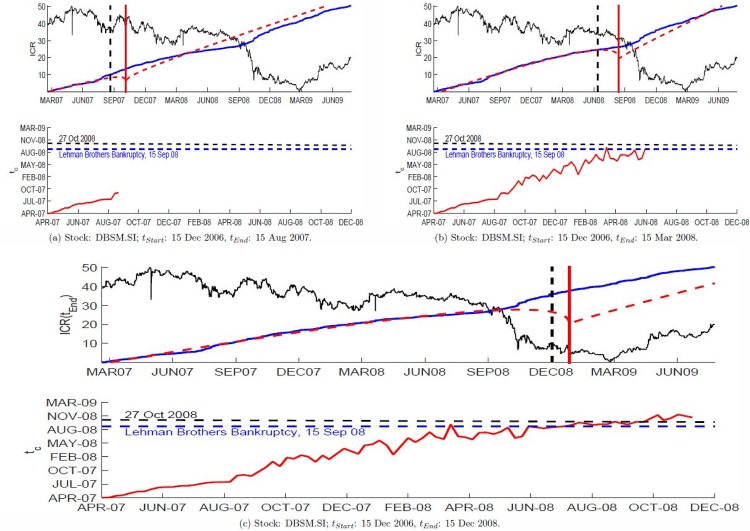
Empirical Integrated Continuous Return *ICR* fitted to the forecasting model $\hat{ICR}$ (Eq (2)). For illustration purposes, we use DBSM.SI and a *t*_*Start*_ of 15 Dec 2006. *t*_*End*_ is then slowly increased with an interval of two weeks. The upper half of each sub-figure shows the empirical *ICR* (blue) and the fitted $\hat{ICR}$ (red dashed-curve), and the rescaled stock price is shown as the background (black). In addition, the black vertical dashed line represents the *t*_*End*_ and red vertical line indicates the corresponding *t*_*c*_. The lower half shows the plot of *t*_*c*_(*t*_*End*_) as a function of *t*_*End*_, where the Lehman Brothers Bankruptcy (15 Sept 2008) is represented by a blue horizontal dashed line and 27 October 2008 is represented by a black horizontal dashed line.

We fix the start *t*_*Start*_ of the fitting window as 15 Dec 2006, which is about two years before the crash. We then slowly expand the fitting window by increasing the *t*_*End*_ of the fitting window. Fig 2(a)–2(c) show a series of fits for *t*_*End*_ as 15 Aug 2007, 15 Mar 2008, and 15 Dec 2008. In these figures, the black vertical line represents *t*_*End*_ and the red vertical line represents the corresponding forecasted crash time *t*_*c*_. In the lower half of each sub-figure, we plot the corresponding *t*_*c*_ against *t*_*End*_ up to *t*_*End*_ as 15 Aug 2007, 15 Mar 2008, and 15 Dec 2008 with 15 Dec 2006 as the *t*_*Start*_. From the graph, we can see that when far before the October 2008 crash, *t*_*c*_ is increasing linearly with *t*_*End*_. This seems to suggest that the SGX is always critical, and ready to crash. This agrees with the suggestions that stock markets are self-organized critical (SOC) systems [30, 31], as well as findings from calibrating agent-based models in stock markets [32].

As we approach the October 2008 crash, we see *t*_*c*_ growing at a slower rate and finally stagnating around the actual crash time (*t*_*Act*_ = 27 Oct 2008). This stagnation occurs a few months before *t*_*End*_ reaches *t*_*Act*_. This is an interesting early warning signature that we must look out for in a system that is already bordering on criticality. This result gives a positive outlook for applying the SOG model to forecast market crashes, but rigorous tests are needed to establish the forecasting power of the SOG model.

## Discussion

**Fig 3:**
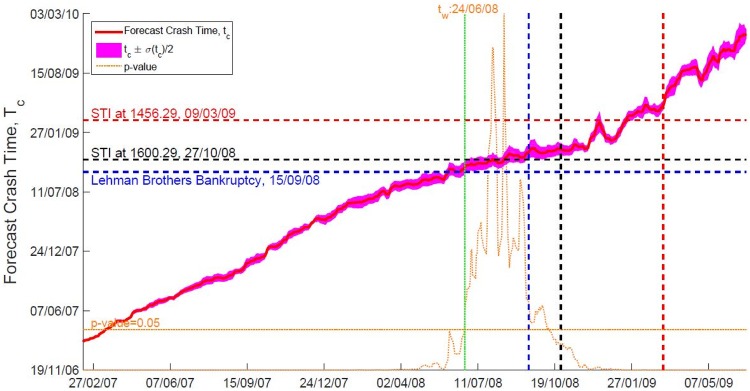
In this figure we show the sensitivity test for stock DBSM.SI, where the blue dashed line represents the date of Lehman Brothers Bankruptcy (15 Sept 2008), black dashed line represents the our benchmark date-27 Oct 2008 when the STI at its lowest due to October 2008 crash, and red dashed line marks the date 9 Mar 2009 that the STI at its lowest due to Asian Correction. We plot the weighted average forecast crash time *t*_*c*_ against *t*_*End*_ showing the weighted standard deviation as the spread. We also show the *p*-value for our null hypothesis *H*_*o*_: *t*_*c*_(*t*_*End*_) = 27 Oct 2008 cutoff at 95% confidence level (orange curve).

We perform a sensitivity test on the forecasting results by comparing the forecasted crash time *t*_*c*_ with the October 2008 crash, actual crash time *t*_*Act*_ = 27 Oct 2008. Given a *t*_*Start*_, *t*_*c*_ is a function of *t*_*End*_ as *t*_*c*_ = *t*_*c*_(*t*_*End*_|*t*_*Start*_). We can test the sensitivity of *t*_*c*_(*t*_*End*_|*t*_*Start*_) to *t*_*End*_ using 14 *t*_*Start*_’s at the beginning and middle of the months from 1 Nov 2006 to 16 May 2007. We compare the weighted mean of forecasted crash time, ${\bar{t}}_{c}\left(t_{End}\right)\text{s}$ with the actual crash time *t*_*Act*_ = 27 Oct 2008 at 95% confidence level, using the null hypothesis $H_{o}:{\bar{t}}_{c}=t_{Act}$ (see the subsection on Sensitivity analysis in Data and Methods section). Robust signatures for the market crash forecast can be observed in Fig 3: ${\bar{t}}_{c}$ increases linearly with *t*_*End*_ when we are far from the market crash, and thereafter stagnates around *t*_*Act*_ when *t*_*End*_ is closer to the October 2008 crash. For earlier *t*_*End*_’s the predicted ${\bar{t}}_{c}$ is statistically different from *t*_*Act*_, whereas for *t*_*End*_ close to the actual crash there is no longer statistical difference between ${\bar{t}}_{c}$ and *t*_*Act*_.

The *t*_*End*_ when *t*_*c*_ first becomes statistically indistinguishable from *t*_*Act*_ is the *warning time*, *t*_*w*_ which in turn acts as the early warning signal for market crashes. In addition, in order to determine the accuracy of the SOG forecasting result, we calculate the average of the weighted mean of forecasted crash time $\langle {\bar{t}}_{c}\left(t_{End}\right)\rangle$: average of ${\bar{t}}_{c}\left(t_{End}\right)$ for the *t*_*End*_’s between the warning time *t*_*w*_ and the actual crash time *t*_*Act*_ (27 Oct 2008). If the $\langle {\bar{t}}_{c}\left(t_{End}\right)\rangle$ calculated is close to *t*_*Act*_, it means the forecasting result is accurate. The results for all 20 component stocks are listed in the S Table 1 in S5 File, there is a range of four to six months of early warning prior the actual crash. Apart from the component stock DBSM.SI with $\langle {\bar{t}}_{c}\left(t_{End}\right)\rangle$ falling on 29 Oct 2008, the others predicted crash dates are after *t*_*Act*_, as late as 12 Feb 2009. This is to be expected, as the SOG model predicts the latest possible crash time. However, in reality it is possible for a giant cluster to disintegrate before it reaches its maximum size.

In addition to the latest possible crash date for individual stocks that we are tracking, we can also combine the forecasting results from all stocks into an index to estimate the market-level risk. For example, when the majority of the STI component stocks predict a crash simultaneously on a particular date, the market risk heightens on that particular date. In order to do this cross sectional study, we first count the number of STI component stocks forecasted to crash on a particular day, for various *t*_*Start*_’s and *t*_*End*_’s. Going back to Fig 2, we observe that when we are far from the actual crash, the forecasted crash date increases linearly with *t*_*End*_ and thus we get a uniform background count. In contrast, when *t*_*End*_ is close to *t*_*Act*_, *t*_*c*_ stagnates leading to a high concentration of forecasted crashes in a narrow range of dates and hence a large number of counts on those dates. We show these counts as a heat map to visualize the market risk, where a date with large counts hence high market risk is shown as a red pixel. In contrast, a blue pixel means background level of counts with low market risk.

**Fig 4:**
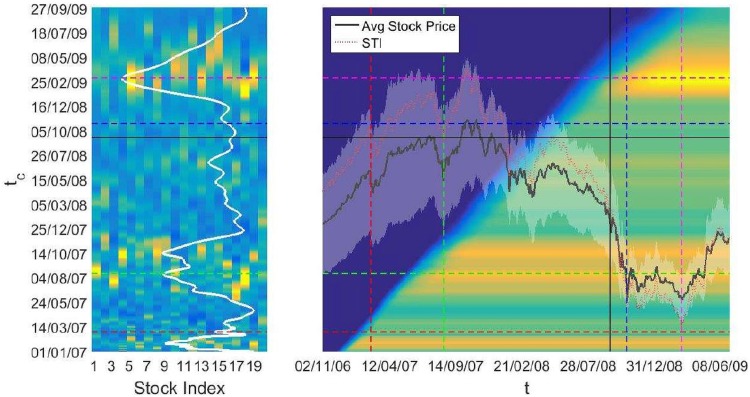
Market risk heat map of the SGX. When a majority of the stocks simultaneously forecast a crash on a particular date, the market risk is high on that date. In this figure, a red pixel indicates a high market risk while the background risk level (low stress) is shown as blue pixels. For both figures, the dash horizontal lines represent the lowest of STI due to: (red) 27 Feb 2007 Chinese Correction, (green) begin of landslide of Subprime Crisis-17 Aug 2007, (blue) Lehman Brothers crisis-9 Oct 2008, (magenta) Asian Correction-9 Mar 2009, and the black solid horizontal line shows the collapse of Lehman Brothers, 15 Sep 2008. [Left] Heat map for all 20 stock with *t*_*End*_ running from Jan 2007 to Jun 2009. The white curve represents the sum of risk level for all stocks, which peaks at 25 Jan 2007, 14 Aug 2007, 14 Oct 2007, and 5 Mar 2009. [Right] Sum of all 20 stocks risk level with *t*_*End*_ running from Jan 2007 to time *t*. The mean and standard deviation of daily price for all stocks is shown as the black curve with white band. The increase in risk level can be seen a few months prior to the actual crash time, and thus can act as an early warning for market crashes.

On the left of Fig 4, we vary *t*_*End*_ over the period Jan 2007 to Jun 2009. For each stock, we accumulate the forecasted crash time *t*_*c*_ into a histogram and these histograms are then represented as a heat map. In principle if we are at time *t*, we will not know the forecasted crash time for *t*_*End*_ > *t*. Thus on the right of Fig 4, we show the average counts for all 20 STI component stocks, but only vary *t*_*End*_ from Jan 2007 up to *t*. We see that there are four red bands for *t*_*c*_ peaking at 25 Jan 2007, 14 Aug 2007, 14 Oct 2007, and 5 Mar 2009. These episodes of heightened market risks are close to the lowest of STI due to the Chinese Correction (27 Feb 2007), begin of the landslide of the Subprime Crisis (17 Aug 2007), and the Asian Correction (9 Mar 2009). However, there is no heightened market risk near the collapse of Lehman Brothers bankruptcy (15 Sep 2008). We believe this is due to the October 2008 crash and the Asian Correction appearing as a single extended market crash to the forecasting model. More interestingly, early warning signals did appear a few months before the October 2008 crash, thus providing us the opportunity to prepare for the financial market crashes.

All in all, these results show that the SOG forecasting model provides early warning in the form of slowing down of the integrated continuous returns. Furthermore the SOG model is also able to indicate the tension level for potential financial crashes via the market risk heat map. Together with other standard critical slowing down indicators like increasing volatility, increasing autocorrelation, red shift in power spectrum, and increasing cross-correlation [33–35], the combination of indicators can act as financial crisis early warning alarm for authorities, such that measures can be taken to prevent or at least soften the impact of financial crashes. Still, there is room for improvement, for example the false negative error for the October 2008 crash compared to Asian Correction. We do not expect that the SOG model to be the best model for describing market fusion-fission dynamics, particularly the complete fragmentation required by the SOG model. We believe partial fragmentation of clusters will be more realistic, and we are working to the test forecasting method based on SOG-like models. In summary, the SOG model forecasting is promising enough to merit further exploration.

## Data and Methods

### Data

In this study, we focus on data from the Singapore Exchange (SGX) because we are familiar with stocks listed in the SGX, in contrast to model markets (e.g. the London Stock Exchange (LSE) and the New York Stock Exchange (NYSE)) that are often studied by econophysicists. Although the SGX is an emerging market, it is strongly coupled to financial markets around the world, and readily reflects the global market movement. For instance, the Straits Times Index (STI) is highly correlated with the Dow Jones Index (*ρ* ≈ 0.91), and slid more than 45% between August 2008 and October 2008 as the result of the October 2008 crash (see S Fig 1 in S1 File). The STI is made up of the top 30 stocks in SGX, but these components do change from time to time. Thus we only consider 20 component stocks that remain highly traded across January 2006 to December 2011. See S Table 1 in S1 File for the full list of these 20 stocks.

The tick-by-tick data was downloaded from the Thomson Reuters Tick History database (http://thomsonreuters.com/tick-history). On an average the number of transactions per stock is on the order of 10^6^ over 1506 trading days. Unlike LSE and NYSE, the SGX is relatively illiquid as the median time interval between transactions ranges from 6 s for the most liquid stock to 47 s for the least liquid stock (refer to S Table 1 in S1 File).

### Continuous returns

We define *continuous return* as the continuous price movements in the same direction, and mathematically the continuous return *CR*_*t*(*m*)_ is given as

$$\begin{array}{c} CR_{t(m)}=\frac{|P_{t(m+n)}-P_{t(m)}|}{P_{t(m)}}, \end{array}$$ (5)

that satisfies all conditions simultaneously

$$\begin{array}{ccc} \left(P_{t(m-1)}-P_{t(m)})\times (P_{t(m+n)}-P_{t(m+n+1)}\right) & > & 0;\\ \left(P_{t(m-1)}-P_{t(m)})\times (P_{t(m)}-P_{t(m+k)}\right) & < & 0;\\ \left(P_{t(m+n)}-P_{t(m+n+1)})\times (P_{t(m+n-k)}-P_{t(m+n)}\right) & < & 0, \end{array}$$

where *P*_*t*(*i*)_ is the *i*^*th*^ transaction price occurs at time *t*(*i*), *k* ∈ 1, 2, 3 … *n*, and at time *t*_*m*+*n*_ the direction of the price movement changes. S Fig 3 in S3 File illustrates a segment of stock price time series, where the continuous return is the fractional price change between two red vertical lines, denoting the start and end of a microtrend [36]. Over the study period, Jan 2006 to Dec 2011 the number of continuous returns on average across stocks is on the order of 10^4^, and the median of microtrend for different stocks ranged from 10 mins to 50 mins (see S Table 1 in S3 File).

### Sensitivity analysis

We perform a sensitivity test of the forecasting results by comparing the weighted mean forecasted crash time ${\bar{t}}_{c}$ with the actual crash time *t*_*Act*_. Given a *t*_*Start*_, *t*_*c*_ is a function of *t*_*End*_ as *t*_*c*_ = *t*_*c*_(*t*_*End*_|*t*_*Start*_). We can test the sensitivity of *t*_*c*_(*t*_*End*_|*t*_*Start*_) to *t*_*End*_, by calculate the weighted mean of *t*_*c*_ at particular *t*_*End*_ using various *t*_*Start*_’s. The weighted mean and weighted standard deviation of *t*_*c*_(*t*_*End*_) is then calculated for each respective *t*_*End*_ as

$$\begin{array}{ccc} {\bar{t}}_{c}\left(t_{End}\right)=\sum_{i=1}^{N}w(t_{Start}^{i}|t_{End})\;t_{c}\left(t_{End}|t_{Start}^{i}\right); & & \\ \sigma^{2}(t_{c}\left(t_{End}\right))=\frac{\sum_{i=1}^{N}w\left(t_{Start}^{i}|t_{End}\right)\;{\left[t_{c}\left(t_{End}|t_{Start}^{i}\right)-{\bar{t}}_{c}\left(t_{End}\right)\right]}^{2}}{N}; & \end{array}$$ (6)

where the weight is defined as

$$\begin{array}{c} w\left(t_{Start}^{i}|t_{End}\right)=\frac{NumDays(t_{Start}^{i},t_{End})}{\sum_{i=1}^{N}NumData\left(t_{Start}^{i},t_{End}\right)}. \end{array}$$

In Eq (6) the *NumDays*(*t*_1_, *t*_2_) is the number of trading days between *t*_1_ and *t*_2_, and weight $w(t_{Start}^{i}|t_{End})$ is introduced to take into account the different amount of data being used to do the forecast. We compare ${\bar{t}}_{c}\left(t_{End}\right)$ with the *t*_*Act*_ at 95% confidence level, using the null hypothesis $H_{o}:{\bar{t}}_{c}=t_{Act}$, where the *t*-value and *p*-value for *n* observations are calculated as

$$\begin{array}{ccc} t_{value}\left(t_{End}\right)=\frac{|{\bar{t}}_{c}\left(t_{End}\right)-t_{Act}|}{\sigma (t_{c}\left(t_{End}\right))}; & & \\ p_{value}\left(t_{End}\right)=1-\int_{-\infty }^{t_{value}\left(t_{End}\right)}\frac{\Gamma (\frac{n}{2})}{\Gamma (\frac{n-1}{2})}\frac{1}{\sqrt{n\pi }}\frac{1}{{\left(1+\frac{x^{2}}{n-1}\right)}^{\frac{n}{2}}}dx, & \end{array}$$ (7)

where Γ(*x*) is the gamma function. When *p* < 0.05, *t*_*c*_ is significantly different from *t*_*Act*_ at 95% confidence level, and we reject *H*_*o*_. Otherwise, we fail to reject *H*_*o*_ when *p*_*value*_ is greater than 0.05.

## Supporting Information

S1 File Supplementary Document: Data, provides the details about data we use in this study. (PDF)

S2 File Supplementary Document: Soup-of-Groups Model, provides the details about the Soup-of-Groups model introduced by Johnson et al. (PDF)

S3 File Supplementary Document: Continuous Returns, explains the details about Continuous Returns and and shows the continuous return distribution fits with PL, EXP, and ETPL distribution. (PDF)

S4 File Supplementary Document: PL, EXP, and ETPL distribution fitting, explains the fitting process for PL, EXP, and ETPL distribution. (PDF)

S5 File Supplementary Document: Warning Time and Average Forecasted Crash Time, indicates the forecasting result for the 20 component stocks. (PDF)

S1 Data Processed Data. We are not at liberty to share raw tick-by-tick data downloaded from the Thomson-Reuter TickHistory (TRTH) database, because of the terms of our subscription. Readers who would like to study the same raw data set that we did should subscribe to the TRTH database. However, we are happy to provide processed data, which includes continuous returns, forecasted crash times corresponding to different *t*_*Start*_ and *t*_*E*_ *nd*, and market risk heat map. These data were uploaded as supplementary document as S1 Data.
(ZIP)
