# Supplementary material for: The Asian Correction Can Be Quantitatively Forecasted Using a Statistical Model of Fusion-Fission Processes
Source: PLoS One. 2016 Oct 5;11(10):e0163842. doi: 10.1371/journal.pone.0163842 (PMC5051705; doi:10.1371/journal.pone.0163842)
Supplement: S1 File — (PDF) [file pone.0163842.s001.pdf]

Financial Market Crashes Can Be Quantitatively Forecasted
Supplementary Document: Data

Boon Kin Teh[a,b,*], Siew Ann Cheong[a,b]

[a] *Division of Physics and Applied Physics, School of Physical and Mathematical Sciences, Nanyang Technological University, 21 Nanyang Link, Singapore 637371, Republic of Singapore*
[b] *Complexity Institute, Block 2 Innovation Centre, Level 2 Unit 245, Nanyang Technological University, 18 Nanyang Drive, Singapore 637723, Republic of Singapore.*

Data

We focus on data from the Singapore Exchange (SGX) in this study because we are more familiar with stocks listed in the SGX, in contrast to model markets (e.g. the London Stock Exchange (LSE) and the New York Stock Exchange (NYSE)) that are often studied by econophysicists. Although the SGX is an emerging market, it is strongly coupled to financial markets around the world, and readily reflect the global market movement. For instance, the Straits Times Index (STI) is highly correlated with Dow Jones Industrial Average Index ($\rho \approx 0.91$),and slid more than 45% between August 2008 and October 2008 as the result of the Lehman Brothers Crisis (see S Fig 1). The STI is made up of the top 30 stocks in SGX, but these components do change from time to time. Thus we only consider 20 component stocks that remain highly traded across January 2006 to December 2011. See S Table 1 for the full list of these 20 stocks.

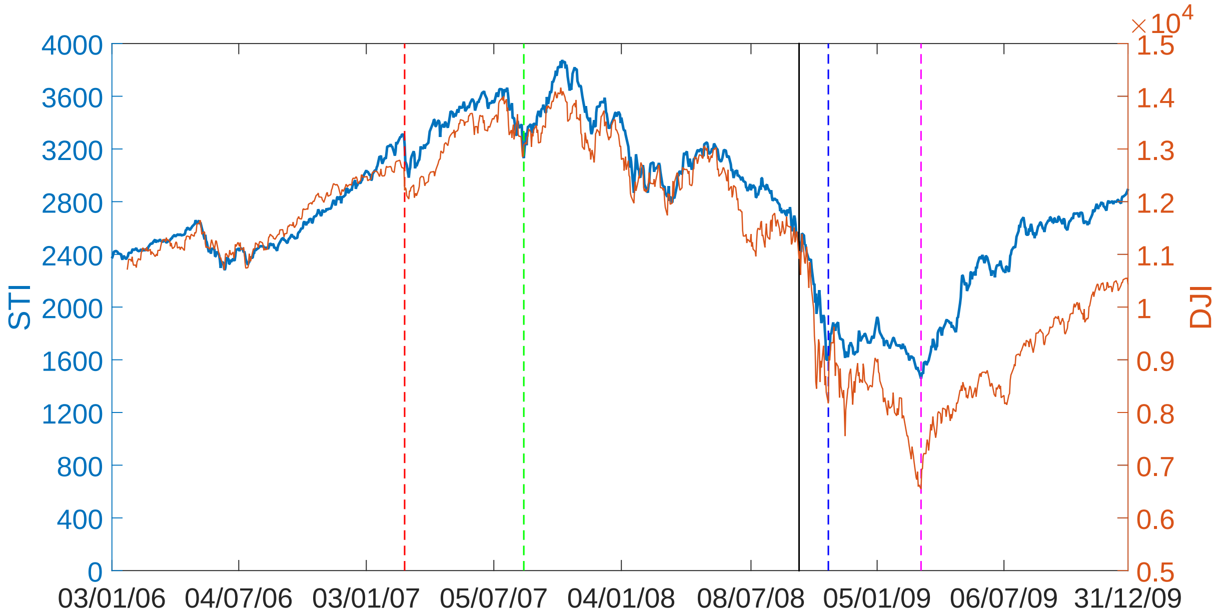

S Fig 1: Straits Times Index (daily) from January 2006 to December 2011 downloaded from Yahoo, Singapore Finance [url:`https://sg.finance.yahoo.com/q/hp?s=%5ESTI`] and Dow Jones Industrial Average Index (daily) downloaded from the Federal Reserve Bank of ST. Louis [url:`https://research.stlouisfed.org/fred2/series/DJIA/downloaddata`]. The correlation between STI and DJI is high ($\rho \approx 0.91$) indicating that the Singapore market is strongly affected by market activities in the United States. The dash vertical lines represent the lowest of STI due to: (red) 27 Feb 2007 Chinese Correction, (green) begin of landslide of Subprime Crisis-17 Aug 2007, (blue) Lehman Brothers crisis-27 Oct 2008, (magenta) Asian Correction-09 Mar 2009, and the black solid vertical line shows the collapse of Lehman Brothers, 15 Sep 2008.

*Corresponding author
Email addresses: `S130005@e.ntu.edu.sg` (Boon Kin Teh), `cheongsa@ntu.edu.sg` (Siew Ann Cheong)

The tick-by-tick data was downloaded from the Thomson Reuters Tick History database (`http://thomsonreuters.com/tick-history`). On average the number of transactions per stock is on the order of 10^6 over 1506 trading days. Unlike LSE and NYSE, the SGX is relatively illiquid, as the median time interval between transactions ranges from 6 s for most liquid stock to 47 s for least liquid stock (see S Table 1). Across the period studied, the STI experience two crashes and tumbled to its lowest at 1600.29 (27 Oct 2008, October 2008 crash), with a slight recovery after that and slid further to 1456.29 (9 Mar 2009, Asian Correction). The first crash was larger thus we select 27 Oct 2008 as our reference date for the market crash.

S Table 1: Stocks codes and transaction details for the 20 stocks studied in this paper. Notes that Q1, Q2, and Q3 represent 25%, 50%, and 75% percentile across all transactions and continuous returns intervals from January 2008 to December 2008.

No	Stock Code	Stock Name	Transactions				Continuous Returns			
			Number	Interval (s)			Number	Interval (s)		
				Q1	Q2	Q3		Q1	Q2	Q3
01	CATL	CapitaLand	1436783	2	7	24	14017	103	679	3756
02	CMDG	ComfortDELGRO	435726	3	21	88	6795	359	2895	9453
03	COSC	Cosco Corp.	1137049	2	8	29	7592	262	2016	7905
04	CTDM	City Development	669882	3	13	52	11115	187	1210	4948
05	DBSM	DBS Bank	1304215	2	7	25	16635	77	492	3393
06	FRNM	Fraser & Neave	529932	3	17	70	8021	338	2249	7237
07	GAGR	Golden Agri	1137261	2	9	29	7799	129	1485	7026
08	HKLD	HongKong Land	547430	2	13	67	11450	233	1289	4775
09	JARD	Jardine Matheson	232975	5	30	149	9249	179	1260	5777
10	JCYC	Jardine C&C	210416	8	47	185	9164	276	1659	5924
11	KPLM	Keppel Corp.	1256012	1	7	26	13097	112	776	3909
12	NOBG	Noble Group	1333328	1	6	23	9376	128	1183	5778
13	OCBC	OCBC Bank	1013364	3	11	37	11852	152	1053	4489
14	SCIL	Sembcorp Industry	719008	2	13	53	8752	286	1928	6604
15	SGXL	Singapore Exchange	1175935	2	9	29	11504	122	918	4637
16	SIAL	Singapore Airline	647313	3	15	58	11526	179	1112	4546
17	STAR	StarHub	367062	4	24	101	7196	417	2940	8735
18	STEL	Singtel	1293144	2	9	29	13287	88	722	3991
19	UOBH	UOB Bank	949453	2	9	36	14016	120	747	3803
20	WLIL	Wilmar	1129634	1	8	29	8996	214	1412	5497
