# Supplementary material for: The Asian Correction Can Be Quantitatively Forecasted Using a Statistical Model of Fusion-Fission Processes
Source: PLoS One. 2016 Oct 5;11(10):e0163842. doi: 10.1371/journal.pone.0163842 (PMC5051705; doi:10.1371/journal.pone.0163842)
Supplement: S2 File — (PDF) [file pone.0163842.s002.pdf]

Financial Market Crashes Can Be Quantitatively Forecasted
Supplementary Document: Soup-of-Groups Model

Boon Kin Teh[a,b,*], Siew Ann Cheong[a,b]

[a] *Division of Physics and Applied Physics, School of Physical and Mathematical Sciences, Nanyang Technological University, 21 Nanyang Link, Singapore 637371, Republic of Singapore*

[b] *Complexity Institute, Block 2 Innovation Centre, Level 2 Unit 245, Nanyang Technological University, 18 Nanyang Drive, Singapore 637723, Republic of Singapore.*

Soup-of-Groups Model

The Soup-of-groups (SOG) model was introduced by Johnson et al. to study emergent behaviors in vastly different complex systems. It was first applied to model the underlying dynamics of human insurgency[1], where terrorists are assumed to form groups that merge with each other in preparation for attacks, and disintegrate after a successful attack or to avoid detection by security forces. Johnson et al. found by comparing the model against a database of past terrorist attacks that the terrorist groups are equally effective, and the number of casualties depends only on the group size, which is distributed as a power law with exponent $\alpha = 2.5$. Following this, Johnson et al. also try to explain contagion dynamics on social network in term of the SOG model, particularly in the intermediate regime where individual behaviors sensitively influence the structures of social groups[2]. In the follow up paper[3] they suggested that the escalation rate and timing of the fatal attacks from terrorist follow naturally from SOG dynamics.

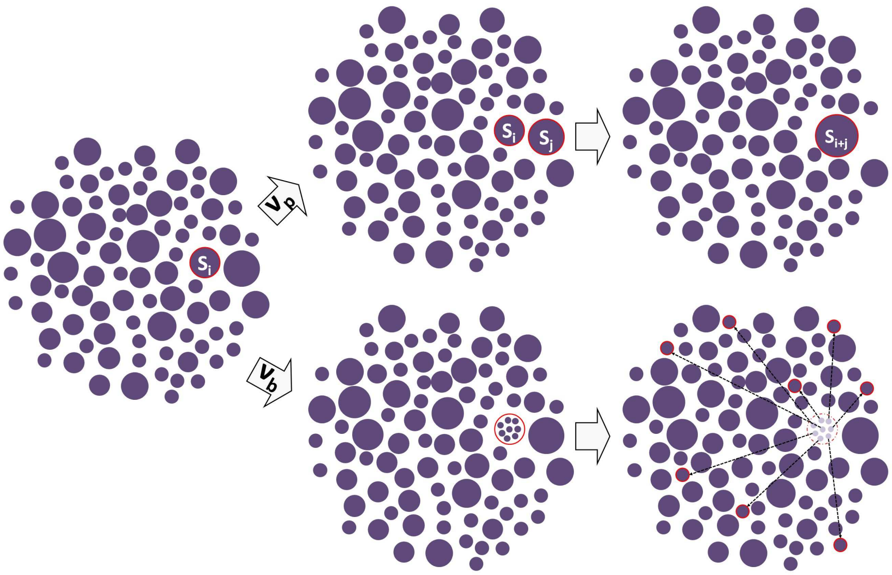

S Fig 1: Illustration of the Soup-of-Groups (SOG) model, as a system of clusters with various sizes, where clusters can experience either fusion or fission with probability v_p or v_b respectively. The upper half represents the fusion process as two clusters with size s_i and s_j are selected and fuse into a larger cluster with size $s_i + s_j$. In contrast, during the fission process (lower half) the selected cluster with size s_i completely fragments into s_i size-1 clusters.

The SOG model, as the name suggests, consists of a "soup" (system) of "groups" (clusters) with sizes ranging from 1 to N, where N is the total number of particles within the system. The essence of SOG dynamics is illustrated in S Fig 1, where a cluster of size s_i can merged with a cluster of size s_j to give a cluster with size $s_i + s_j$ at a rate

*Corresponding author
Email addresses: S130005@e.ntu.edu.sg (Boon Kin Teh), cheongsa@ntu.edu.sg (Siew Ann Cheong)

of ν_p, or a cluster of size s_k can fragment completely into s_k clusters with size-1 at a rate of ν_b. These fusion-fission processes can be described by the master equation

$$\begin{aligned} \frac{\partial N_1}{\partial t} &= \dot{N}_1 = \nu_b \sum_{k=2}^{\infty} k \frac{k}{N} N_k - 2\nu_p \frac{1}{N} N_1 \sum_{k=1}^{\infty} \frac{k}{N} N_k \\ \frac{\partial N_s}{\partial t} &= \dot{N}_s = \nu_p \sum_{k=2}^{s-1} \frac{k}{N} N_k \frac{s-k}{N} N_{s-k} - 2\nu_p \frac{s}{N} N_s \sum_{k=1}^{\infty} \frac{k}{N} N_k - \nu_b \frac{s}{N} N_s; \quad s \geq 2. \end{aligned} \tag{1}$$

When the SOG system is at equilibrium, all $\dot{N}_i = 0$, allowing Neil Johnson et al. to solve for the equilibrium distribution, which is an *Exponentially Truncated Power Law* (ETPL)[4]

$$N_s = f(S) \sim s^{-\frac{5}{2}} \exp(-S_o s); \quad S_o = -\ln\left(\frac{4(\nu_p+\nu_b)\nu_p}{(2\nu_p+\nu_b)^2}\right). \tag{2}$$

References

[1] Juan Camilo Bohorquez, Sean Gourley, Alexander R Dixon, Michael Spagat, and Neil F Johnson. Common ecology quantifies human insurgency. *Nature*, 462(7275):911–914, 2009.

[2] Zhenyuan Zhao, JP Calderón, Chen Xu, Guannan Zhao, Dan Fenn, Didier Sornette, Riley Crane, Pak Ming Hui, and Neil F Johnson. Effect of social group dynamics on contagion. *Physical Review E*, 81(5):056107, 2010.

[3] Neil Johnson, Spencer Carran, Joel Botner, Kyle Fontaine, Nathan Laxague, Philip Nuetzel, Jessica Turnley, and Brian Tivnan. Pattern in escalations in insurgent and terrorist activity. *Science*, 333(6038):81–84, 2011.

[4] Neil F Johnson, Josef Ashkenazi, Zhenyuan Zhao, and Luis Quiroga. Equivalent dynamical complexity in a many-body quantum and collective human system. *AIP Advances*, 1(1):012114, 2011.
