# Supplementary material for: The Asian Correction Can Be Quantitatively Forecasted Using a Statistical Model of Fusion-Fission Processes
Source: PLoS One. 2016 Oct 5;11(10):e0163842. doi: 10.1371/journal.pone.0163842 (PMC5051705; doi:10.1371/journal.pone.0163842)
Supplement: S3 File — (PDF) [file pone.0163842.s003.pdf]

Financial Market Crashes Can Be Quantitatively Forecasted Supplementary Document: Continuous Returns

Boon Kin Teh[a,b,*], Siew Ann Cheong[a,b]

[a]*Division of Physics and Applied Physics, School of Physical and Mathematical Sciences, Nanyang Technological University, 21 Nanyang Link, Singapore 637371, Republic of Singapore*

[b]*Complexity Institute, Block 2 Innovation Centre, Level 2 Unit 245, Nanyang Technological University, 18 Nanyang Drive, Singapore 637723, Republic of Singapore.*

Continuous Returns

Definition

In finance, one of the most widely used definition for quantifying financial returns is the simple return, $r_t = \frac{P_t - P_{t-1}}{P_{t-1}}$ and log return, $Lr_t = \log(1 + r_t)$. Stylized facts have been discovered, for instance the distribution of the return (log return) tend to have heavy tail for short intervals (less than a day) but approach the normal distribution for long intervals[1]. The choice of the time interval is therefore ambiguous. Because of this ambiguity, we introduce the *continuous returns* defined as the continuous prices movement in the same direction. Mathematically the continuous return ($CR_{t(m)}$) is given by

$$CR_{t(m)} = \frac{\mid P_{t(m+n)} - P_{t(m)} \mid}{P_{t(m)}}, \tag{1}$$

satisfying simultaneously

$$\begin{aligned} (P_{t(m-1)} - P_{t(m)}) \times (P_{t(m+n)} - P_{t(m+n+1)}) &> 0; \\ (P_{t(m-1)} - P_{t(m)}) \times (P_{t(m)} - P_{t(m+k)}) &< 0; \\ (P_{t(m+n)} - P_{t(m+n+1)}) \times (P_{t(m+n-k)} - P_{t(m+n)}) &< 0, \end{aligned}$$

where $P_{t(i)}$ is the ith transaction price at time $t(i)$, $k \in 1, 2, 3...n$, and at time t_{m+n} the direction of the price movement changes.

This definition of an event agrees with that used by da Cruz and Lind[2]. In financial markets, market participants are heterogeneous, so though they read the same market signals they may arrive at different trading strategies. However, if the market signal is strong, then the traders will arrive at the same trading strategies thereby reinforcing the market signals, leading to a formation of strategy blocks. When a strategy block is executed, market participants within the strategy block will react in the same way, hence generating a continuous return with a magnitude that depends on the size of the strategy block. S Fig 1 illustrates a segment of stock price time series, where continuous return is the factional price change between two red vertical lines, which denote the start and end a microtrend[3]. Over the study period, Jan 2006 to Dec 2011 the number of continuous returns on average across stocks is on the order of 10^4, and the median of microtrend for different stocks ranged from 10 mins to 50 mins (see S Table 1).

*Corresponding author

Email addresses: `S130005@e.ntu.edu.sg` (Boon Kin Teh), `cheongsa@ntu.edu.sg` (Siew Ann Cheong)

S Table 1: Stocks codes and transaction details for the 20 stocks studied in this paper. Notes that Q1, Q2, and Q3 represent 25%, 50%, and 75% percentile across all transactions and continuous returns intervals from January 2008 to December 2008.

No	Stock Code	Stock Name	Transactions Number	Transactions Interval (s) Q1	Q2	Q3	Continuous Returns Number	Continuous Returns Interval (s) Q1	Q2	Q3
01	CATL	CapitaLand	1436783	2	7	24	14017	103	679	3756
02	CMDG	ComfortDELGRO	435726	3	21	88	6795	359	2895	9453
03	COSC	Cosco Corp.	1137049	2	8	29	7592	262	2016	7905
04	CTDM	City Development	669882	3	13	52	11115	187	1210	4948
05	DBSM	DBS Bank	1304215	2	7	25	16635	77	492	3393
06	FRNM	Fraser & Neave	529932	3	17	70	8021	338	2249	7237
07	GAGR	Golden Agri	1137261	2	9	29	7799	129	1485	7026
08	HKLD	HongKong Land	547430	2	13	67	11450	233	1289	4775
09	JARD	Jardine Matheson	232975	5	30	149	9249	179	1260	5777
10	JCYC	Jardine C&C	210416	8	47	185	9164	276	1659	5924
11	KPLM	Keppel Corp.	1256012	1	7	26	13097	112	776	3909
12	NOBG	Noble Group	1333328	1	6	23	9376	128	1183	5778
13	OCBC	OCBC Bank	1013364	3	11	37	11852	152	1053	4489
14	SCIL	Sembcorp Industry	719008	2	13	53	8752	286	1928	6604
15	SGXL	Singapore Exchange	1175935	2	9	29	11504	122	918	4637
16	SIAL	Singapore Airline	647313	3	15	58	11526	179	1112	4546
17	STAR	StarHub	367062	4	24	101	7196	417	2940	8735
18	STEL	Singtel	1293144	2	9	29	13287	88	722	3991
19	UOBH	UOB Bank	949453	2	9	36	14016	120	747	3803
20	WLIL	Wilmar	1129634	1	8	29	8996	214	1412	5497

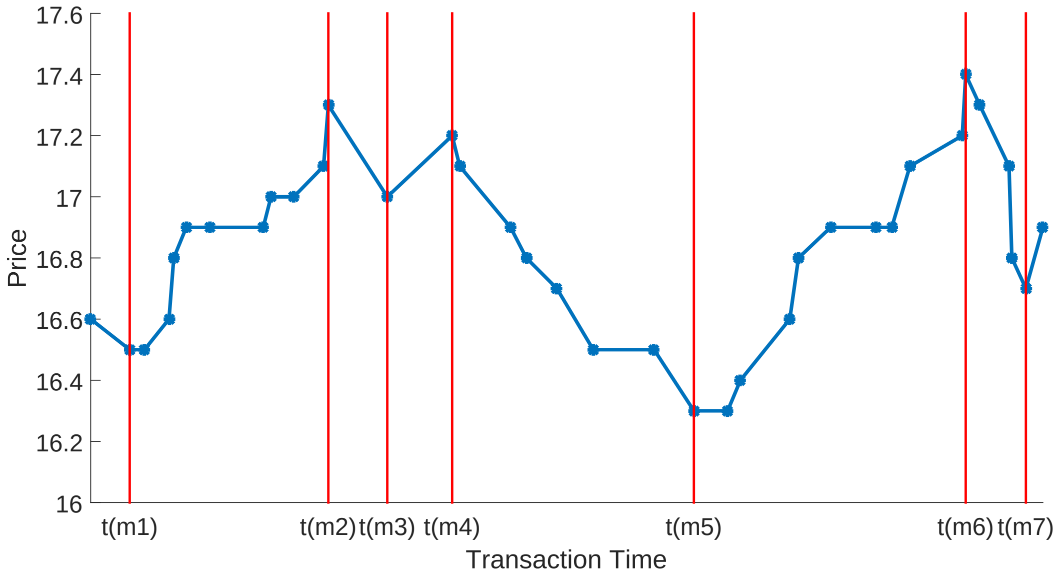

S Fig 1: A segment of stock price movement that illustrates the definition of a *continuous return*. A continuous return is a series of price movements in the same direction. In this figure, each point represents a transaction price and the time interval is different as transactions can occur at any time. Each vertical line indicates the transaction time when the price movement changes direction. Thus, each vertical line is considered as a continuous return $CR_{t(m_i)}$ with magnitude defined as $CR_{t(m_i)} = |P_{t(m_{i+1})}) - P_{t(m_i)}|/P_{t(m_i)}$. Refer to Equation (1) for the details.

Distribution of Continuous Returns.

The SOG dynamics is universal, so we expect to be able to do forecasting with it even when the stock price dynamics is only approximately SOG. To demonstrate this approximate SOG behavior we check the distribution of the continuous return against ETPL distribution. For comparison we also fit the empirical data with power law (PL) and asymptotic exponential (EXP) distributions. Details of the fitting procedure can be found in S4 File. The fitting results indicate that the continuous returns for 20 stocks are best fitted by the ETPL. In S Fig 2 we show (a) a typical, (b) good, and (b) poor set of fits to the ETPL, PL, and EXP. When the fit is poor non of the distributions fit the continuous returns well. Fortunately there are few poor fits. The full fitting results are shown in Appendix A. Overall, the average α_{ETPL} and β_{ETPL} across all stocks is 1.82 ± 0.23 and 0.37 ± 0.08 respectively.

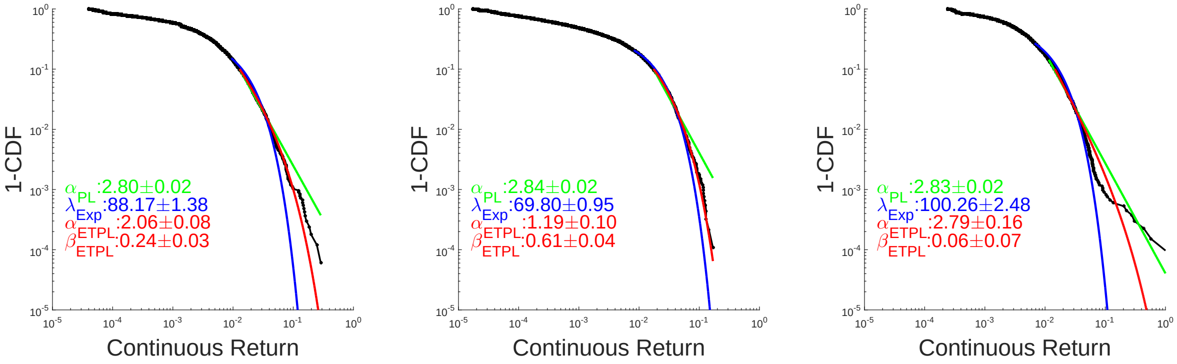

(a) Typical fit, DBSM.SI **(b)** Good fit, JARD.SI **(c)** Poor fit, STEL.SI

S Fig 2: Fits of continuous return distribution (black) with the exponential truncated power law, $ETPL$ (red) benchmarked against the power law, PL (green) and asymptotic exponential, EXP (blue) distributions. In (a) typical and (b) good fits, the ETPL fits continuous returns the best, while the best PL decays too slowly and the best EXP decays too quickly. However, in (c) poor fits none of the distributions fit the data well.

References

[1] Cont R. Empirical properties of asset returns: stylized facts and statistical issues. Quantitative Finance. 2001;1(2):223–236.

[2] Da Cruz JP, Lind PG. Self-organized criticality in a network of economic agents with finite consumption. Physica A: Statistical Mechanics and its Applications. 2012;391(4):1445–1452.

[3] Preis T, Schneider JJ, Stanley HE. Switching processes in financial markets. Proceedings of the National Academy of Sciences. 2011;108(19):7674–7678.

Appendix A. Continuous Return Distribution Fitting Results

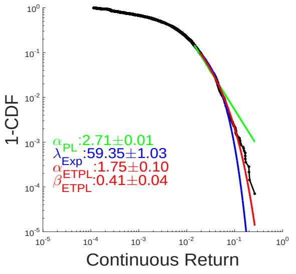

(a) CATL

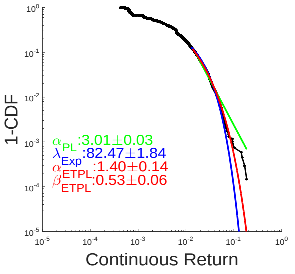

(b) CMDG

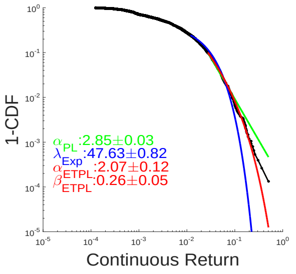

(c) COSC

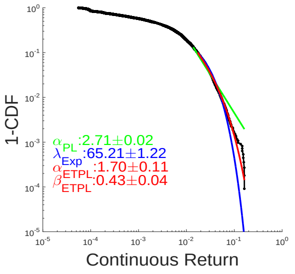

(d) CTDM

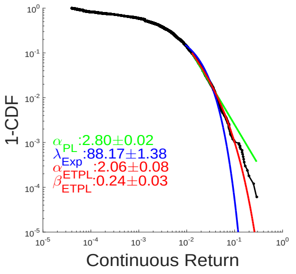

(e) DBSM

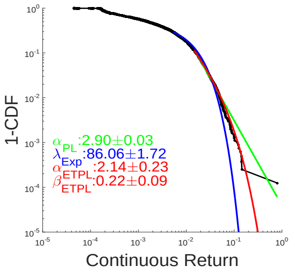

(f) FRNM

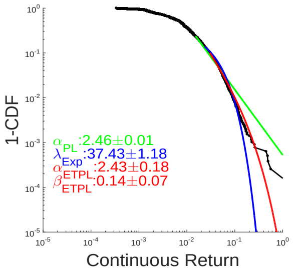

(g) GAGR

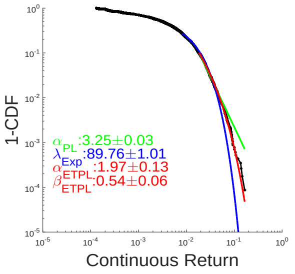

(h) HKLD

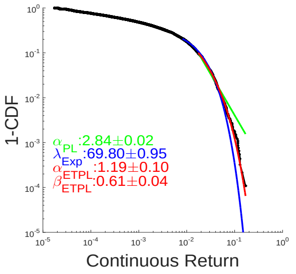

(i) JARD

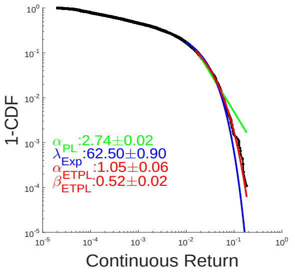

(j) JCYC

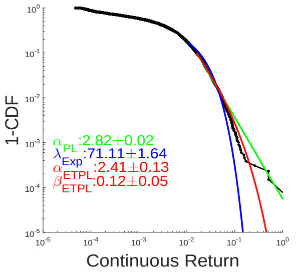

(k) KPLM

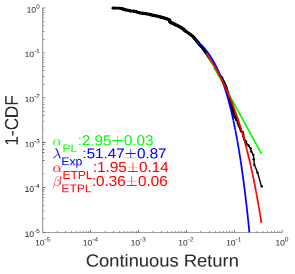

(l) NOBG

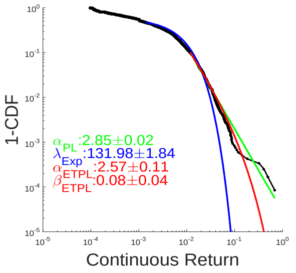

(m) OCBC

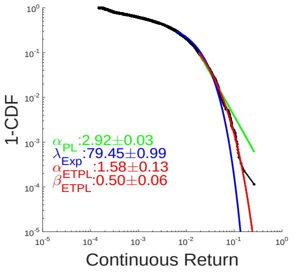

(n) SCIL

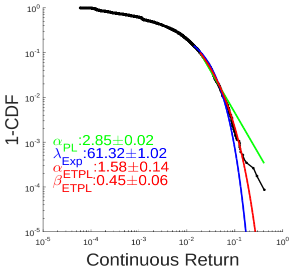

(o) SGXL

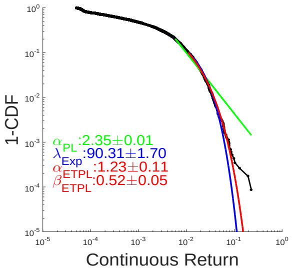

(p) SIAL

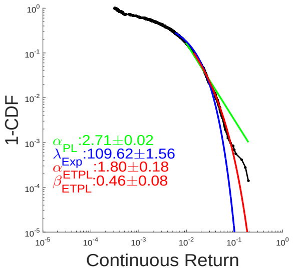

(q) STAR

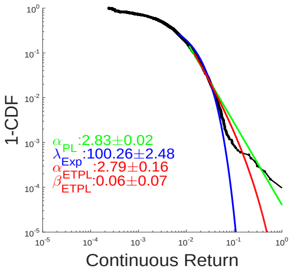

(r) STEL

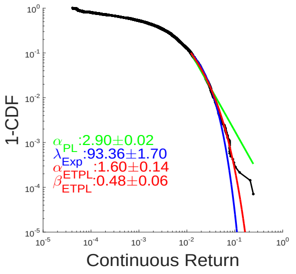

(s) UOBH

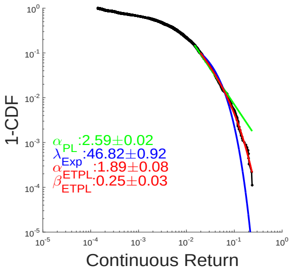

(t) WLIL
