# Supplementary material for: The Asian Correction Can Be Quantitatively Forecasted Using a Statistical Model of Fusion-Fission Processes
Source: PLoS One. 2016 Oct 5;11(10):e0163842. doi: 10.1371/journal.pone.0163842 (PMC5051705; doi:10.1371/journal.pone.0163842)
Supplement: S4 File — (PDF) [file pone.0163842.s004.pdf]

Financial Market Crashes Can Be Quantitatively Forecasted
Supplementary Document: PL, EXP, and ETPL Distribution Fitting

Boon Kin Teh[a,b,*], Siew Ann Cheong[a,b]

[a] *Division of Physics and Applied Physics, School of Physical and Mathematical Sciences, Nanyang Technological University, 21 Nanyang Link, Singapore 637371, Republic of Singapore*

[b] *Complexity Institute, Block 2 Innovation Centre, Level 2 Unit 245, Nanyang Technological University, 18 Nanyang Drive, Singapore 637723, Republic of Singapore.*

PL, EXP, and ETPL Distribution Fitting

Given a data set $\boldsymbol{X}$ to fit to a power law (PL), asymptotic exponential (EXP), and exponential truncated power law ($ETPL$) distributions, we use the method proposed by Clauset et al. to estimate the parameters[1]. Except for x_{min}, the remaining parameters are estimated using the maximum likelihood method. The normalized distribution and likelihood functions of PL, EXP, and $ETPL$ distributions for domain $x \in [x_{min}, \infty)$ are listed in S Table 1. The estimation of x_{min} is done by measuring the goodness of fit (Gof) upon fitting truncated data ($\boldsymbol{X} > x_{temp}$) to a particular distribution. The x_{temp} that minimized the Gof is chosen as the estimator of x_{min}.

The Gof is calculated as the Kolmogorov–Smirnov distance between $F(\boldsymbol{X})$ and the uniform distribution, where F is the cumulative distribution function for $\boldsymbol{X}$. Kolmogorov–Smirnov distance is one of the non-parametric tools used to compare two distribution functions, by measuring the maximum distance between two cumulative distribution functions. This is because $F(\boldsymbol{X})$ is a uniform distribution function range between 0 and 1 if the cumulative distribution function of $\boldsymbol{X}$ is indeed F. Mathematically, the goodness of fit Gof is defined as

$$\text{KS-D} = \max\left|CF\left(F(\boldsymbol{x})\right) - CF\left(U(\boldsymbol{x})\right)\right|, \tag{1}$$

where $CF(.)$ means cumulative distribution function.

S Table 1: The normalized distribution function of power law (PL), asymptotic exponential (EXP), and exponential truncated power law ($ETPL$) distributions for domain $x \in [x_{min}, \infty)$. In addition, their likelihood functions also given which will be used to estimate the parameter set via the maximum likelihood. In this table, $\Gamma(1-\alpha,\beta)$ represents the incomplete gamma function defined as $\Gamma(1-\alpha,\beta) = \int_\beta^\infty x^{-\alpha}\exp(-x)dx$.

Type	Distribution, $x \in [x_{min}, \infty)$	Likelihood Function
$f_{PL}(x\|\alpha)$	$\frac{\alpha-1}{{x_{min}}^{1-\alpha}}x^{-\alpha}$	$N\ln(\hat{\alpha}-1) + (\hat{\alpha}-1)N\ln(x_{min}) - \hat{\alpha}\sum_{i=1}^N \ln(x_i)$
$f_{EXP}(x\|\lambda)$	$\lambda\exp\{-\lambda(x-x_{min})\}$	$N\ln(\hat{\lambda}) - \hat{\lambda}\sum_{i=1}^N x_i - x_{min}$
$f_{ETPL}(x\|\alpha,\beta)$	$\frac{\beta^{1-\alpha}}{x_{min}\Gamma(1-\alpha,\beta)}\left(\frac{x}{x_{min}}\right)^{-\alpha}\exp\left(-\frac{\beta x}{x_{min}}\right)$	$N\ln\left\{\frac{\hat{\beta}^{1-\hat{\alpha}}}{x_{min}\Gamma(1-\hat{\alpha},\hat{\beta})}\right\} - \hat{\alpha}\sum_{i=1}^N \ln\left\{\frac{x_i}{x_{min}}\right\} - \hat{\beta}\sum_{i=1}^N \frac{x_i}{x_{min}}$

References

[1] Aaron Clauset, Cosma Rohilla Shalizi, and Mark EJ Newman. Power-law distributions in empirical data. *SIAM Review*, 51(4):661–703, 2009.

*Corresponding author

Email addresses: S130005@e.ntu.edu.sg (Boon Kin Teh), cheongsa@ntu.edu.sg (Siew Ann Cheong)
