# Supplementary material for: The Asian Correction Can Be Quantitatively Forecasted Using a Statistical Model of Fusion-Fission Processes
Source: PLoS One. 2016 Oct 5;11(10):e0163842. doi: 10.1371/journal.pone.0163842 (PMC5051705; doi:10.1371/journal.pone.0163842)
Supplement: S5 File — (PDF) [file pone.0163842.s005.pdf]

Financial Market Crashes Can Be Quantitatively Forecasted
Supplementary Document: Warning Time and Average Forecasted Crash Time

Boon Kin Teh[a,b,*], Siew Ann Cheong[a,b]

[a]*Division of Physics and Applied Physics, School of Physical and Mathematical Sciences, Nanyang Technological University, 21 Nanyang Link, Singapore 637371, Republic of Singapore*

[b]*Complexity Institute, Block 2 Innovation Centre, Level 2 Unit 245, Nanyang Technological University, 18 Nanyang Drive, Singapore 637723, Republic of Singapore.*

Warning Time and Average Forecasted Crash Time

In the main text, we perform a sensitivity test of the forecasting results by comparing the forecasted crash time t_c with the actual October 2008 crash t_{Act} =27 Oct 2008. In order to do this, we fit the empirical integrated continuous return with the forecasting model using various t_{Start}'s to obtain a distribution of t_c for a particular t_{End}. The weighted average $\bar{t}_c(t_{End})$ and weighted standard deviation σ^2 $(t_c(t_{End}))$ are then being calculated. We compare $\bar{t}_c(t_{End})$ with t_{Act} at 95% confidence level with the null hypothesis as $H_o : \bar{t}_c = t_{Act}$. When the $p < 0.05$, t_c is significantly different from t_{Act} at 95% confidence level, and we reject H_o. Otherwise, we fail to reject H_o when p-value is greater than 0.05.

S Table 1: The warning time, t_w that act as the precursor for for market crash and average forecasted crash time $\langle \bar{t}_c \rangle$ for the 20 STI component stocks studied. Compared with the actual market crash, t_{Act} (27 Oct 2008), the early warning is ranged from four to six months prior the actual crash time, while the average forecasted crash time $\langle \bar{t}_c \rangle$ range from 29 Oct 2008 to 12 Feb 2009. In this Table TDs stand for trading days.

**No.**	**Index**	t_w	$\langle \bar{t}_c \rangle$	**No.**	**Index**	t_w	$\langle \bar{t}_c \rangle$
01	CATL	15/05/2008	07/01/2009±17 TDs	11	CMDG	02/06/2008	04/12/2008±16 TDs
02	COSC	21/04/2008	13/01/2009±21 TDs	12	CTDM	07/05/2008	08/01/2009±19 TDs
03	DBSM	03/06/2008	29/10/2008±09 TDs	13	FRNM	27/05/2008	15/12/2008±13 TDs
04	GAGR	15/04/2008	12/02/2009±25 TDs	14	HKLD	26/05/2008	05/12/2008±13 TDs
05	JARD	26/05/2008	06/01/2009±21 TDs	15	JCYC	02/05/2008	19/12/2008±14 TDs
06	KPLM	25/06/2008	10/12/2008±13 TDs	16	NOBG	30/04/2008	30/01/2009±22 TDs
07	OCBC	04/06/2008	28/11/2008±12 TDs	17	SCIL	12/05/2008	11/12/2008±16 TDs
08	SGXL	12/05/2008	02/01/2009±16 TDs	18	SIAL	20/05/2008	15/12/2008±14 TDs
09	STAR	20/05/2008	17/12/2008±14 TDs	19	STEL	14/05/2008	18/11/2008±08 TDs
10	UOBH	30/05/2008	24/11/2008±12 TDs	20	WLIL	12/05/2008	30/01/2009±28 TDs

The sensitivity test for all 20 component stocks are shown in Appendix A. Robust signatures for the market crash forecast can be observed, as t_c increased linearly with t_{End} when we are far from the market crash, and thereafter stagnated around t_{Act} when t_{End} is closer to the October 2008 crash. For earlier t_{End}'s the predicted t_c is statistically different from t_{Act}, whereas for t_{End} close to the actual crash there is no longer statistical difference between t_c and t_{Act}. We call t_w, the t_{End} when t_c first becomes statistically indistinguishable from t_{Act} the *warning time*. We also calculate the average forecasted crash time $\langle \bar{t}_c(t_{End}) \rangle$ using t_{End}'s between t_w and t_{Act} (27 Oct 2008). If the $\langle \bar{t}_c(t_{End}) \rangle$ calculated is close to t_{Act}, it means the forecasting result is accurate. The results for all 20 stocks are listed in S Table 1. There is four to six months of early warning prior the actual crash, and apart from DBSM.SI, whose $\langle \bar{t}_C(t_{End}) \rangle$ falls on 29 Oct 2008, the others predicted crash dates after t_{Act}, as late as 12 Feb 2009.

*Corresponding author

Email addresses: `S130005@e.ntu.edu.sg` (Boon Kin Teh), `cheongsa@ntu.edu.sg` (Siew Ann Cheong)

Appendix A. Forecasting Results

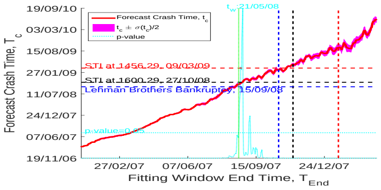

(a) CATL

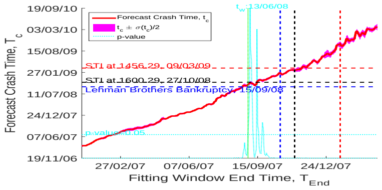

(b) CMDG

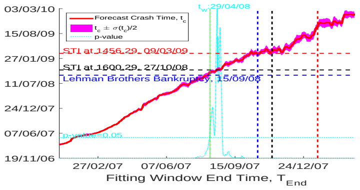

(c) COSC

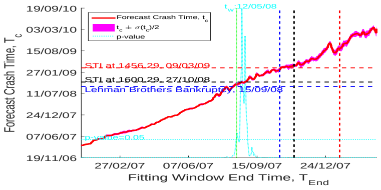

(d) CTDM

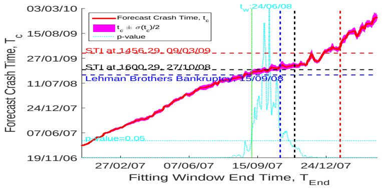

(e) DBSM

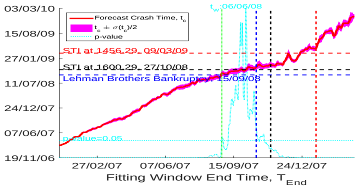

(f) FRNM

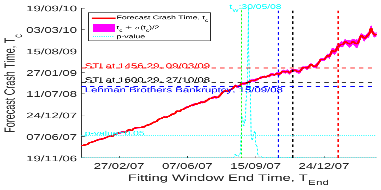

(g) GAGR

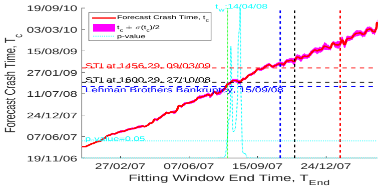

(h) HKLD

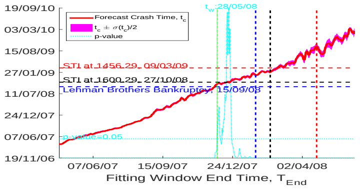

(i) JARD

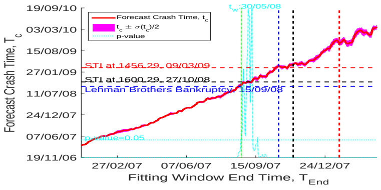

(j) JCYC

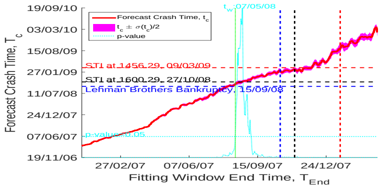

(k) KPLM

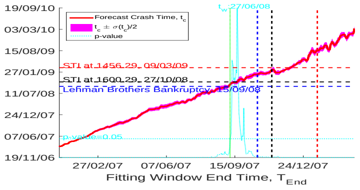

(l) NOBG

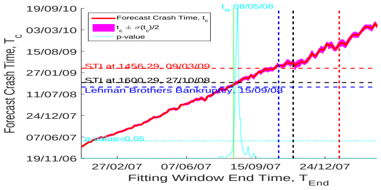

(m) OCBC

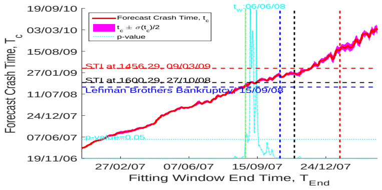

(n) SCIL

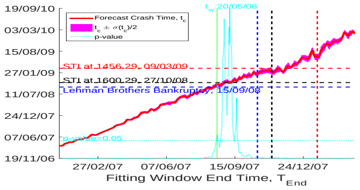

(o) SGXL

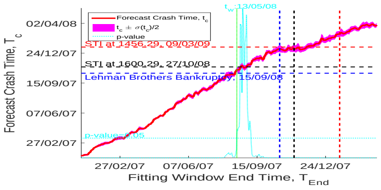

(p) SIAL

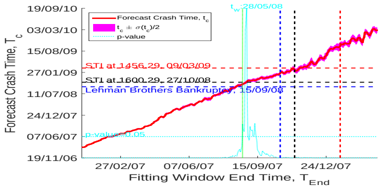

(q) STAR

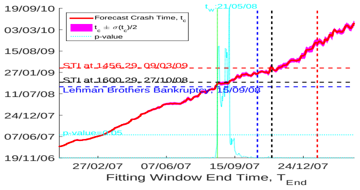

(r) STEL

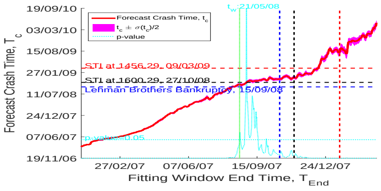

(s) UOBH

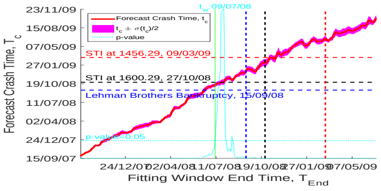

(t) WLIL
