# Supplementary figures and images for: The Asian Correction Can Be Quantitatively Forecasted Using a Statistical Model of Fusion-Fission Processes
Source: PLoS One. 2016 Oct 5;11(10):e0163842. doi: 10.1371/journal.pone.0163842 (PMC5051705; doi:10.1371/journal.pone.0163842)

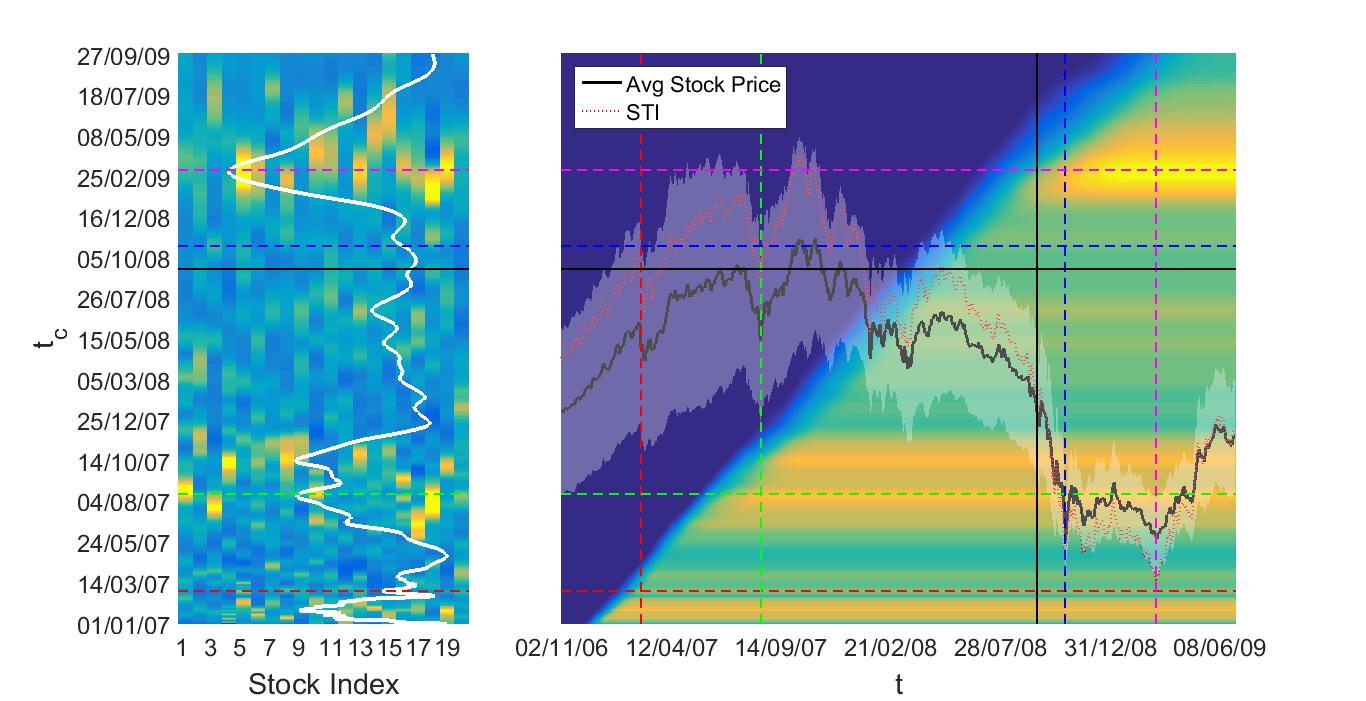

Supplement: S1 Data — We are not at liberty to share raw tick-by-tick data downloaded from the Thomson-Reuter TickHistory (TRTH) database, because of the terms of our subscription. Readers who would like to study the same raw data set that we did should subscribe to the TRTH database. However, we are happy to provide processed data, which includes continuous returns, forecasted crash times corresponding to different tStart and tE nd, and market risk heat map. These data were uploaded as supplementary document as S1 Data. (ZIP) [file pone.0163842.s006.zip › S_Data/MarketRiskHeatMap/HeatMap.jpg]
